# To assess the confidence levels of psychiatrists in physical healthcare competencies in one irish region, and to explore whether confidence was related to learning opportunities

**DOI:** 10.1192/j.eurpsy.2021.1587

**Published:** 2021-08-13

**Authors:** M. O’Donnell, V. Pradeep, C. Dunne, G. Gulati, B. Kelly

**Affiliations:** 1 Midwest Region, Health Service Executive, Limerick, Ireland; 2 Dublin, St John Of Gods, PW, Ireland; 3 University Of Limerick, School of Medicine, Limerick, Ireland

**Keywords:** Education and Training, Co-morbidities, Physical health, Outcome studies

## Abstract

**Introduction:**

The bi-directional relationship between mental and physical illness is well established. Therefore, in order to lower the already high mortality rates associated with psychiatric disorders, physical health issues must be closely monitored in this population [1,2]. A recent Lancet commission highlights emerging strategies and recommendations for improvement of physical health outcomes in patients with chronic mental disorders. These strategies involve better integration of physical and mental health care, combined with broader implementation of lifestyle interventions to reduce elevated cardiometabolic risk and attenuate medication side-effects [3].

**Objectives:**

To assess psychiatrists’ confidence levels in physical healthcare competencies; to explore whether confidence was related to learning opportunities.

**Methods:**

Physical healthcare learning objectives were extracted from the Irish College of Psychiatrists’ training curriculum. An electronic questionnaire was sent to 50 psychiatrists in one Irish healthcare region with a catchment area of c. 450,000. Participants had to rate confidence levels for each competency on a five-point Likert scale and the availability of learning opportunities for attaining each competency.

**Results:**

66% response rate was achieved. A majority reported confidence in cardiovascular examination, interpreting blood results and evaluating comorbidities. A minority reported confidence in interpreting imaging, electrocardiograms and recognising medical emergencies. This corresponds to a relative paucity of learning opportunities.

**Conclusions:**

Clinical implication Programmes for trainee doctors and CME opportunities for consultant psychiatrists would benefit from an emphasis on physical health examination and modules on interpreting investigations and the recognition of medical emergencies.

